# Analysis of Nipah Virus Replication and Host Proteome Response Patterns in Differentiated Porcine Airway Epithelial Cells Cultured at the Air–Liquid Interface

**DOI:** 10.3390/v15040961

**Published:** 2023-04-13

**Authors:** Martin Müller, Kerstin Fischer, Elisabeth Woehnke, Luca M. Zaeck, Christoph Prönnecke, Michael R. Knittler, Axel Karger, Sandra Diederich, Stefan Finke

**Affiliations:** 1 Institute of Molecular Virology and Cell Biology, Friedrich-Loeffler-Institut, Federal Research Institute of Animal Health, 17493 Greifswald-Insel Riems, Germany; 1000000147.gast@fli.de (M.M.); 1000000191.gast@fli.de (E.W.); l.zaeck@erasmusmc.nl (L.M.Z.); axel.karger@fli.de (A.K.); 2 Institute of Novel and Emerging Infectious Diseases, Friedrich-Loeffler-Institut, Federal Research Institute of Animal Health, 17493 Greifswald-Insel Riems, Germany; kerstin.fischer@fli.de (K.F.); sandra.diederich@fli.de (S.D.); 3 Centre for Biotechnology and Biomedicine, Molecular Biological-Biochemical Processing Technology, Leipzig University, 04103 Leipzig, Germany; christoph.proennecke@bbz.uni-leipzig.de; 4 Institute of Immunology, Friedrich-Loeffler-Institut, Federal Research Institute of Animal Health, 17493 Greifswald-Greifswald-Insel Riems, Germany; michael.knittler@fli.de

**Keywords:** Nipah virus, air–liquid interface culture, respiratory epithelium, mass spectrometry, immune response

## Abstract

Respiratory tract epithelium infection plays a primary role in Nipah virus (NiV) pathogenesis and transmission. Knowledge about infection dynamics and host responses to NiV infection in respiratory tract epithelia is scarce. Studies in non-differentiated primary respiratory tract cells or cell lines indicate insufficient interferon (IFN) responses. However, studies are lacking in the determination of complex host response patterns in differentiated respiratory tract epithelia for the understanding of NiV replication and spread in swine. Here we characterized infection and spread of NiV in differentiated primary porcine bronchial epithelial cells (PBEC) cultivated at the air–liquid interface (ALI). After the initial infection of only a few apical cells, lateral spread for 12 days with epithelium disruption was observed without releasing substantial amounts of infectious virus from the apical or basal sides. Deep time course proteomics revealed pronounced upregulation of genes related to type I/II IFN, immunoproteasomal subunits, transporter associated with antigen processing (TAP)-mediated peptide transport, and major histocompatibility complex (MHC) I antigen presentation. Spliceosomal factors were downregulated. We propose a model in which NiV replication in PBEC is slowed by a potent and broad type I/II IFN host response with conversion from 26S proteasomes to immunoproteasomal antigen processing and improved MHC I presentation for adaptive immunity priming. NiV induced cytopathic effects could reflect the focal release of cell-associated NiV, which may contribute to efficient airborne viral spread between pigs.

## 1. Introduction

Bat-borne zoonotic Nipah virus (NiV) causes severe respiratory and neurological disease in pigs and humans [1,2,3]. Together with Hendra virus (HeV) it is eponymous for the genus henipavirus [4]. Since its first emergence in 1998 outbreaks in Bangladesh and India have occurred almost yearly [5,6,7,8]. Genetic characterization of NiV revealed two distinct variants, NiV Malaysia and Bangladesh [9]. Efficient infection of the respiratory tract of pigs is considered causative for the high contagiosity in these hosts and their unique role as intermediate hosts in transmission from the Pteropus bat reservoir to humans [10].

Primary human bronchial epithelial cells (HBEC) are susceptible to NiV infection and react with pro-inflammatory responses [11]. However, infection of fully differentiated HBEC cultivated at the air–liquid interface (ALI), as models for human airway epithelia, results in efficient virus replication and shedding, while only limited host responses are triggered. Accordingly, the trachea-bronchial tropism is considered a determinant of henipavirus contagiosity and efficient human-to-human transmission [12].

Inefficient type I IFN induction with simultaneous pro-inflammatory cytokine expression in NiV-infected, non-differentiated primary porcine bronchial epithelial cells (PBEC) may explain the high susceptibility of the swine respiratory tract to NiV infection [13]. The correlation between susceptibility and limited host responses is corroborated by similar observations in HBEC-ALI cultures [12]. In human and animal cell models, an unbalanced immune response following NiV infection has been postulated [14], which may affect antiviral responses and antigen presentation. In contrast, replication in ferret lungs induces type I IFNs at early phases of infection, indicating that virus replication in the respiratory tract occurs in the context of a potent IFN response and that susceptibility of the respiratory tract tissues does not solely depend on the suppression of innate immune responses by the virus [15].

Henipaviral antagonists of innate immune responses are the phosphoprotein (P) as well as the P variants V, W, and C protein, the matrix protein (M), and the nucleoprotein (N) (reviewed in [14]). For example, the main targets are the signal transducer and activator of transcription (STAT)-1/2 heterodimer [16,17,18], recognition of pathogen-associated molecular patterns (PAMP) [19,20], or tripartite motif-containing 6 (TRIM6)-mediated activation of the inhibitor of nuclear factor kappa B kinase subunit epsilon (IKKε) dependent type I IFN induction and response [21].

Since the respiratory epithelium acts as a barrier of the respiratory tract to NiV entry and at the same time as a significant determinant of virus shedding and airborne transmission, we investigated the infection, spread, and host protein dynamics of NiV in PBEC-ALI cultures. Despite the high burden of viral respiratory infections on public health and the advantages that ALI cultures offer concerning modeling in vivo-like lung tissue in vitro, few articles have been published where high-resolution mass spectrometry (MS)-based proteomics has been applied to virus infected ALI cultures as most of them have focused on SARS-CoV-2 infections [22,23,24,25,26,27]. In the present study, we combined confocal-based imaging with quantitative MS-based proteomics to analyze the dynamics of NiV infection in a differentiated respiratory epithelium ALI model. Virus replication and host reaction patterns were monitored over 12 days. In the context of low or incomplete innate immune responses in porcine and primary cell culture models of other hosts [12,13] and contradictory observations in animal models [15], we hypothesized that host responses to NiV infection in PBEC ALI cultures differ concerning the activation of IFN responses and expression of immunomodulatory genes to those observed in other species or non-differentiated porcine respiratory tract epithelial cells.

Based on real time quantitative PCR (qRT-PCR) analysis of selected host factors and label-free quantitative MS-based proteomics, we demonstrated the regulation of multiple host pathways throughout the infection. We showed robust type I and II IFN responses and broad up-regulation of antigen processing and presentation pathways. The finding of substantial cytopathic effects in the respiratory epithelium may translate into transmission of the cell-associated virus by exhalation or coughing in vivo.

## 2. Materials and Methods

### 2.1. PBECs

PBECs were isolated from two distinct pigs aged around 6 to 12 months. Isolation and cultivation were modified from previous descriptions for other species [28]. Briefly, the cells were prepared from the bronchial epithelium of porcine lungs and 5 × 10^4^ of the cells were transferred to 0.33 cm^2^ cell culture inserts with PneumaCult-Ex medium (STEMCELL Technologies, Köln, Germany) in the basal and apical compartment. When confluency was reached, the apical medium was removed, and the cells were cultivated at the air–liquid interface for at least four weeks. For detailed description see Supplementary Material S1.

### 2.2. Infection of Differentiated PBEC-ALI Cultures

The cultures were washed with PBS three-times before infection with 2 × 10^6^ NiV plaque forming units/well. Then 350 µL inoculum or medium (mock) was added to the apical side. After 1 h, the inoculum was removed, and the apical side was washed three times with PBS. Finally, the medium in the basal compartment was replaced by fresh medium. Cells were incubated for the respective periods, and the medium in the basal compartment was exchanged every 2–3 days. Samples for titration of apically released virus were generated by adding 200 µL of PBS to the apical side and incubating the cells for 30 min at 37 °C and 5% CO_2_. Infectious virus titers in the basal compartment were determined by titration of respective samples.

### 2.3. Virus

NiV (NiV Malaysia, GenBank accession no AF212302) was propagated and titrated on Vero 76 cells (CCLV-RIE 0929, Collection of Cell Lines in Veterinary Medicine (CCLV), Friedrich-Loeffler-Institut, Greifswald, Germany) by plaque assay as described previously with slight modifications [29]. Work with live NiV was performed at the BSL4-facility of the Friedrich-Loeffler-Institut.

### 2.4. Reverse Transcription and Real-Time PCR

RNA extraction was performed with the RNeasy kit (Qiagen, Hilden, Germany) following a DNAse I digest with the TURBO DNA-free Kit (Invitrogen, Waltham, MA, USA). Reverse transcription was performed with 500 ng of RNA using the RevertAid First Strand cDNA Synthesis Kit (Thermo Fisher, Waltham, MA, USA) and Oligo (dt)18 primer. Real-time PCR was carried out with 0.7 µL cDNA product according to the protocol for the PowerUp SYBR Green Master Mix (Thermo Fisher, Waltham, MA, USA), with standard cycling mode (primer Tm ≥ 60 °C). For gene specific primers see Supplementary Material S2. Ct values were normalized to α-tubulin expression (ΔCT). For relative quantification of NiV N mRNA in PBEC-ALI, the ΔCt value was subtracted from the mock control (ΔCt mock − ΔCt infected)

### 2.5. Immunofluorescence and Microscopy

PBECs were fixed for 24 h with 4% paraformaldehyde (PFA) before transfer from the BSL-4 to the BSL-2 facility. After another 24 h fixation, the inserts were washed three times with PBS and stored in PBS + 0.02% sodium azide at 4 °C. For staining of complete membranes, samples were permeabilized with 0.2% Triton X-100 in PBS and blocked with 10% donkey serum/0.3 M glycine/PBST (PBST: 0.1% Tween 20 in PBS). Primary antibodies were incubated for 2 h at room temperature (RT) in 1% donkey serum/PBST following three washing steps with PBS and secondary antibody incubation for 1 h at RT in 1% donkey serum/PBST. For cross-sections, the membranes were removed from the cell culture inserts with a scalpel, paraffin-embedded, and cut into 5 µm microtome slices (HM 430 E, Thermo Scientific). After 15 min permeabilization with 0.2% Triton X-100 in PBS and blocking with 5% donkey serum in PBS TX100, primary antibodies were added for 1 h at RT in 1% donkey serum/PBS TX100. After three washing steps, secondary antibodies were added for 1 h at RT in 1% donkey serum/PBST. Nuclei were stained with Hoechst 33342 (1:20,000 in PBS; Invitrogen, Waltham, MA, USA). For used antibodies see Supplementary Material S2. Images were acquired with a Leica THUNDER imager DMi8 with a 10×/0.32 HC PL FLUOTAR or 20×/0.40 dry HC PL FLUOTAR L objectives and LAS X (v3.7.423463) software, and with a Leica DMI6000 TCS SP5 (Leica Microsystems, Wetzlar, Germany) confocal laser scanning microscope with a 63×/1.40 oil immersion HCX PL APO objective and LAS AF (v2.7.3.9723) software. Images were processed with the Fiji 1.53c software [30]. For scanning electron microscopy (SEM) cells were washed with 0.1 M sodium cacodylate buffer (pH 7.4) and prefixed with 2.5% glutaraldehyde (in 0.1 M sodium cacodylate buffer). Following three washing steps in the same buffer, the cells were post-fixed in 1% OsO4 (0.1 M sodium cacodylate buffer) for 2 h. After three additional washing steps, the cells were dehydrated in an ascending series of ethanol and dried under critical endpoint drying using a Leica EM CP300 (Leica Microsystem, Wetzlar, Germany). For image acquisition, the samples were stuck to stubs, sputter-coated, and analyzed using a SEM (Zeiss EVO LS10).

### 2.6. Preparation and Measurement of MS Samples

Protein extracts from TriFast peqGOLD samples (1 mL TriFast per trans-well) were prepared according to the manufacturer’s instructions and processed as described in detail in Supplementary Material S1. Samples were analyzed on a TimsTOF Pro instrument (Bruker, Germany). The mass spectrometry data were deposited at the ProteomeXchange Consortium via the PRIDE [31] partner repository with the dataset identifier PXD032673 and 10.6019/PXD032673. A detailed description of data processing and analysis is also provided in Supplementary Material S1.

## 3. Results

### 3.1. PBEC-ALI Cultures

**Figure 1:**
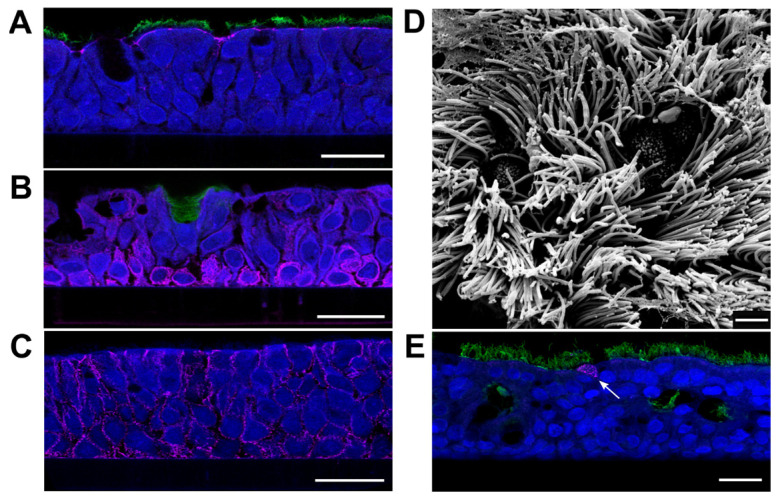
Characterization of PBEC-ALI cultures using cell markers and electron microscopy and susceptibility to NiV infection. (**A**) Immunostaining of cross sections for β-tubulin (ciliated cells; green) and ZO-1 (tight junctions; magenta), (**B**) mucin-5AC (mucus secreting cells; green) and cytokeratin 5 (basal cells; magenta), and (**C**) β-catenin (cell adherens junctions; magenta). (**D**) Scanning electron microscopy visualization of fully-differentiated PBEC-ALI culture after 4 weeks of ALI cultivation. (**E**) Immunostaining for β-tubulin (green) and NiV nucleoprotein N (magenta) at 2 dpi. Arrow: NiV infected cell. Scale bars: 20 µm (**A**–**C**,**E**) and 2 µm (**D**).

Differentiated PBECs were generated by cultivation at the air–liquid interface. Four weeks after seeding in cell culture inserts, generation of 30- to 60 µm-thick differentiated epithelia was confirmed by the appearance of markers for apical tight junctions and cilia (ZO-1 and ß-tubulin; Figure 1A), basal and mucus-secreting cells (cytokeratin 5 and mucin-5AC; Figure 1B), and adherens junctions (β-catenin, Figure 1C). The presence of ciliated and non-ciliated cells at the apical layer was visualized by scanning electron microscopy (SEM) (Figure 1D). Susceptibility of the PBEC-ALI cultures to NiV infection was tested by inoculation with 2 × 10^6^ plaque forming units (pfu)/well and immunofluorescence analysis after 2 days post-infection (dpi). Detection of individual NiV infected cells (Figure 1E) indicated a rather inefficient infection of the PBEC-ALI culture.

### 3.2. Time Course of NiV Infection in PBEC-ALI Cultures

**Figure 2:**
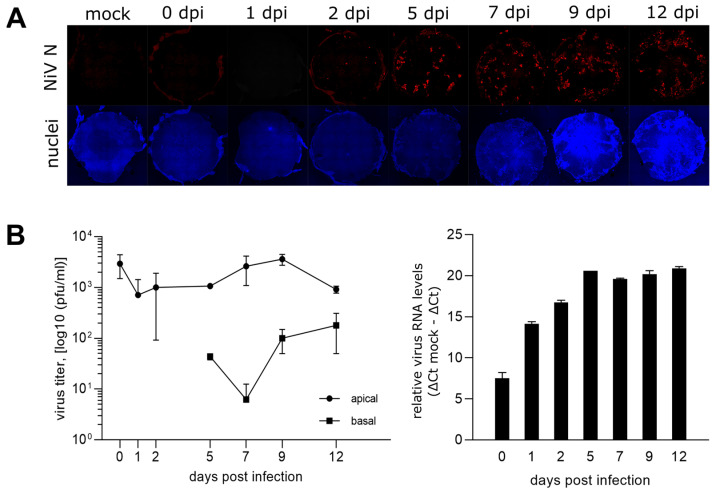
Time course of infected PBEC-ALI cells and virus release. (**A**) Immunostaining of NiV-infected PBEC-ALI membranes against NiV N (red). Nuclei were stained with Hoechst 33342. The diameter of the membranes was approximately 0.64 cm. (**B**) NiV infectious titers at the apical and basal sides. Results are the mean of two replicates with the minimum and maximum values indicated by error bars. On the right, quantification of viral RNA in ALI cultures with NiV N gene primers. The relative virus RNA levels are shown after subtracting ΔCt values from the ΔCt mock value. N = 2. Error bars indicate the min/max values.

To monitor the NiV infection dynamics, the PBEC-ALI cultures were infected with 2 × 10^6^ NiV pfu per well. Cell culture inserts were analyzed by immunofluorescence at 0, 1, 2, 5, 7, 9, and 12 dpi (Figure 2A). Whereas NiV N was not detected at 0 and 1 dpi, NiV N positive spots became visible after 2 dpi. The infection spread further until 12 dpi. However, a complete culture infection was not observed during the 12 days incubation period. Overall, limited numbers of NiV-infected cells at early time points after infection indicated a low specific susceptibility of PBEC-ALI culture from the apical side.

Low amounts of infectious NiV were released at the apical side of the PBEC-ALI cultures, as infectious titers in apical PBS washes remained at input virus levels, determined directly after infection (0 dpi). Even less infectious virus was detected in the basal compartment, where virus titers of 44 to 180 pfu/mL were determined from day 5 on (Figure 2B).

In contrast to the limited virus release at the basal and apical sides, virus replication and spread in the PBEC-ALI cultures was demonstrated by increasing levels of virus RNA (Figure 2B). Together, these data indicated that most newly synthesized NiV remained cell associated.

### 3.3. Lateral NiV Spread and CPE

**Figure 3:**
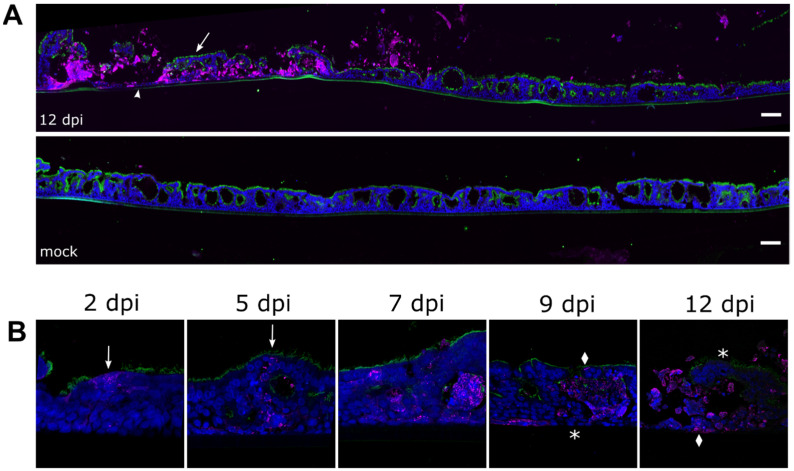
Time course of NiV infection and cytopathic effects. Immunostaining of NiV-infected PBEC-ALI culture cross sections against NiV N (magenta) and β-tubulin (green). (**A**) A large membrane section from 12 dpi at the top and the mock control at the bottom. Arrow and arrowhead indicate less infected and destructed areas of ciliated and basal cells, respectively. Scale bars = 100 µm. (**B**) Time course of NiV infection for 12 dpi with representative sections. Arrows indicate an infection in ciliated and non-ciliated cells of the apical cell layer. Diamonds mark infected cells and stars non-infected cells. Nuclei were stained with Hoechst 33342.

Cross-sections of NiV-infected PBEC-ALI cultures at 12 dpi revealed large-infected areas with a robust cytopathic effect (CPE), disruption of the epithelium, and apical release of virus antigen-positive cell detritus (Figure 3A, top left). Despite the abundance of NiV N protein in infected areas and supernatant cell detritus, the infected areas were separated from the surrounding non-infected areas (Figure 3A, top right). Moreover, in infected and disrupted areas, clusters of ciliated apical and basal cell layers (Figure 3A, arrow and arrowhead, respectively) with less virus antigen were detected when compared with the middle cell layers.

Nevertheless, infection of both ciliated and non-ciliated apical and basal cells at 2 dpi (Figure 3B, arrows) confirmed the general susceptibility of these cell types to NiV infection. Lateral spread of the virus infection was observed for 5 to 12 dpi between the apical and basal cell layers. While increasing cell destruction and cell debris accumulation were observed for the intermediate cell layer, apical and basal cells in these foci were either not infected (Figure 3B, asterisks) or appeared intact in spite of infection (Figure 3B, diamonds). Areas with fragmented cell nuclei were framed by infected or non-infected ciliated apical cell layers and basal cells even at 9 and 12 dpi. These data indicated a mainly lateral cell-to-cell spread of the virus in the PBEC-ALI middle cell layers and that apical and basal cell layers exhibit less CPE.

**Figure 4:**
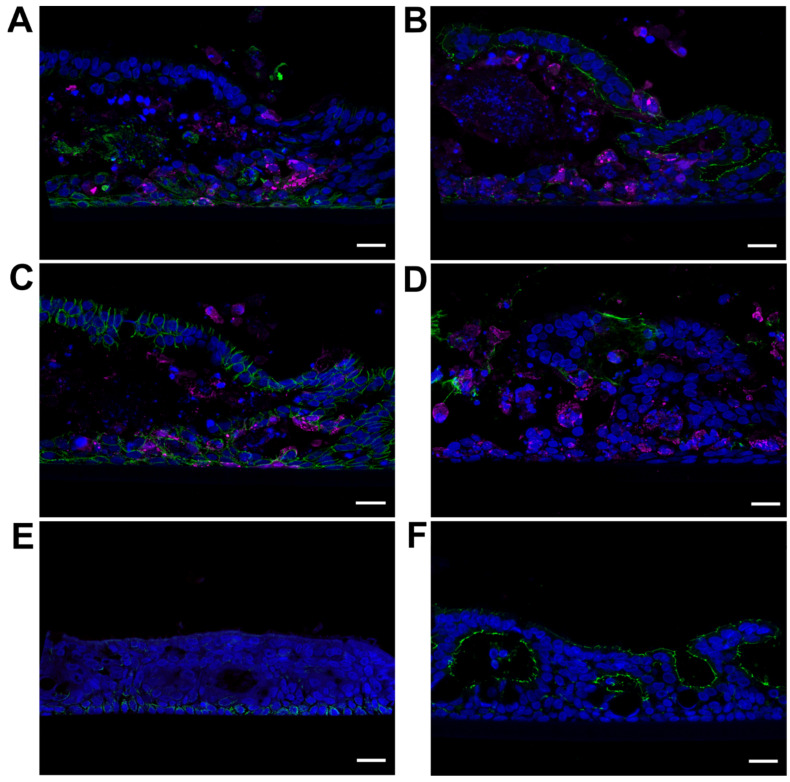
Visualization of NiV cell tropism in cross-sections of infected PBEC-ALI cultures from 12 dpi with various cell markers. (**A**) Immunostaining of a cross section against NiV N (magenta) and cytokeratin 5 as a basal cell marker (green), (**B**) ZO-1 for tight junctions (green), (**C**) β-catenin for adherens junctions (green), and (**D**) mucin-5AC for mucus-secreting cells (green). (**E**) Immunostaining of 12 dpi uninfected areas against NiV N (red) and cytokeratin 5 (green) (**F**) and ZO-1 for tight junctions (green). Nuclei were stained with Hoechst 33342. Scale bars: 20 µm (**A**–**F**).

The structural integrity of the apical and basal cell layers was demonstrated by immunostaining for cytokeratin 5, ZO-1, β-catenin, and mucin-5AC at 12 dpi. A continuous cytokeratin 5 positive layer of infected and non-infected cells below a largely destroyed middle cell layer with condensed chromatin confirmed the integrity of the basal cell layer during NiV infection (Figure 4A). The integrity of the apical tight junctions was demonstrated by ZO-1 stain (Figure 4B). Intact adherens junctions in both apical and basal cell layers were indicated by the β-catenin stain (Figure 4C). Mucin-5AC was detected in apical parts of the epithelium (Figure 4D). Notably, and in contrast to non-infected PBEC-ALI (see Figure 1), beyond areas of strong CPE, ZO-1 appeared at double layered cell packages with tight-junctions at both sides (Figure 4C). Additionally, β-catenin revealed a double-cell layer organization with intact lateral adherens junctions and intact cell nuclei on top of accumulating cell detritus. For comparison, non-infected regions from the same 12 dpi samples were stained for cytokeratin 5 (Figure 4E) and ZO-1 (Figure 4F).

### 3.4. Upregulation of Type I Interferons and Inflammatory Cytokines

**Figure 5:**
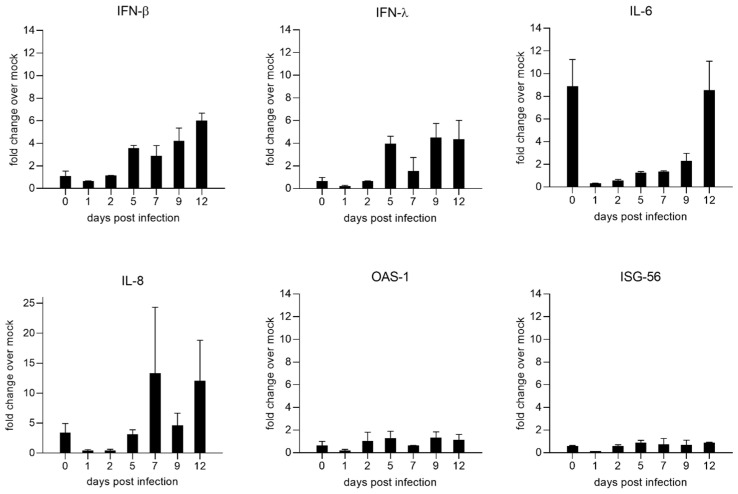
Time course of NiV induced cytokine and IFN-stimulated-gene (ISG) upregulation. Quantifying cytokine and ISG mRNAs by qPCR for IFN-β, IFN-λ, IL-6, IL-8, OAS-1, and ISG-56. Results are depicted as fold change over mock from the mean of two replicates with the value range. Fold change over mock was calculated with the 2^−ΔΔCT^ method.

NiV induced cytokine expression was assessed at 0, 1, 2, 5, 7, 9, and 12 dpi by qRT-PCRs for IFN beta (IFN-β), IFN lambda (IFN-λ), interleukin 6 (IL-6), interleukin 8 (IL-8), oligoadenylate synthetase (OAS-1), and IFN-stimulated gene 56 (ISG-56) (Figure 5). Whereas cytokine expression was low at early time points of infection (days 1 and 2), increased type I (IFN-β) and III IFN (IFN-λ) induction were observed starting at 5 dpi. In contrast to the IFN mRNAs, those of the IFN-stimulated genes OAS-1 and ISG-56 remained at a low level, although these ISG mRNAs also exhibited a week increase from day 1 to 2. Notably, increased levels of IL-6 and IL-8 mRNAs at 0 dpi indicate upregulation of host responses directly after infection. Since these responses returned to basal levels on day 1, these either represented a direct response to the addition of NiV or indicated that the initial rise could be a short-term effect induced by the transport of the cultures to the BSL-4 facility.

### 3.5. NiV Protein Dynamics—Increasing Virus Protein Accumulation until 12 Dpi

**Figure 6:**
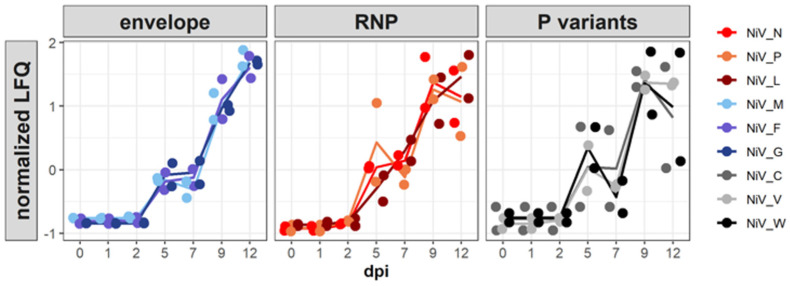
Time course of NiV protein expression. NiV protein levels are shown as normalized values based on label-free quantification. For better visualization, the NiV envelope proteins (M, F, and G), RNP-associated proteins (N, P, and L), and P variants (C, V, and W) are shown in separate panels.

To monitor the virus protein levels and modulations of the host proteomes, protein samples from all time points were subjected to high-resolution MS and quantification based on label-free quantification (LFQ). Given the low initial number of infected cells at 1 dpi (Figure 2), levels of virus proteins at that time point remained close to the level of input proteins detected at 0 dpi (Figure 6). Slightly increased NiV N and P levels at 2 dpi (Figure 6), indicated the onset of NiV gene expression. Further increases in N, P, and L levels at 5 dpi and detection of the envelope M, G, and F proteins correlated with replication and spread of the virus observed by immunofluorescence microscopy and qRT-PCR detection (Figure 2). At late time points (9 and 12 dpi), all virus proteins, including accessory V, W and C proteins were detected. Both replicates of the infection kinetics revealed a robust increase in structural virus protein levels over time. These data confirmed the time course of N expression by immunofluorescence (Figure 2) on the proteome level in a quantitative and reproducible manner.

### 3.6. Variation of the Host Cell Proteome during Infection

**Figure 7:**
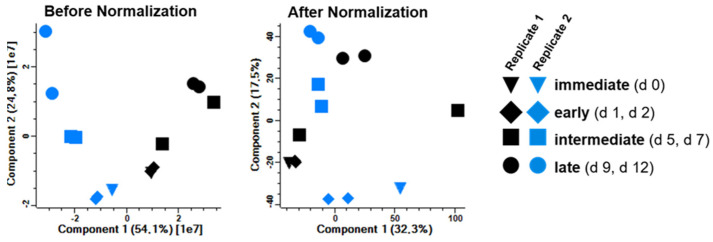
Principal component analysis (PCA). Quantitative MS data of both replicates were subjected to PCA analysis before and after normalization. Before normalization, sample clustering is dominated by animal-to-animal variability. Normalization partially compensated for these effects and resulted in a more time-dependent clustering of immediate, early, intermediate, and late samples from both animals.

In both infection time course experiments, a total of 6345 host proteins were identified by MS and considered for further statistical analysis. The principal component analysis (PCA) of the quantitative data (Figure 7, left panel) indicated the time-dependent clustering of samples. A dominant influence of the cell cultures from the different animals on the composition of the proteomes could be partially compensated by the normalization of the quantitative data (Figure 7, right panel), resulting in a more time-dependent clustering of all samples.

In a first statistical evaluation of all time points, the expression kinetics of all identified proteins (virus and host) were subjected to a von Neumann test [32] to detect proteins showing a time dependent trend in their expression levels. Proteins with *p*-values < 0.05 in both replicates of the kinetic experiment were subjected to GO analysis with the gProfiler R-package. The resulting enriched GO terms (see Table S4) strongly suggested activation of the innate immune response (GO:0045087) and other pathways.

### 3.7. DEGs and GO Term Enrichment Analysis

For the identification of differentially expressed genes (DEG) and further statistical evaluation of the impact of NiV infection on the host proteome the early (1 dpi, 2 dpi) and late (9 dpi, 12 dpi) stages were selected for comparison. This selection was based on the clustering behavior of these samples in the PCA analysis (Figure 7), the fact that 5 dpi and 7 dpi represented a transition phase concerning the expression of viral proteins (Figure 6), and that qPCR analysis of the day 0 samples (Figure 5) suggested that these may be artificial.

Statistical testing (left or right-sided *t*-test, *p*-value < 0.05) of normalized data identified 730 upregulated and 123 downregulated genes (Table S1) at the late stage of infection when compared to the early stage of infection as DEG.

**Figure 8:**
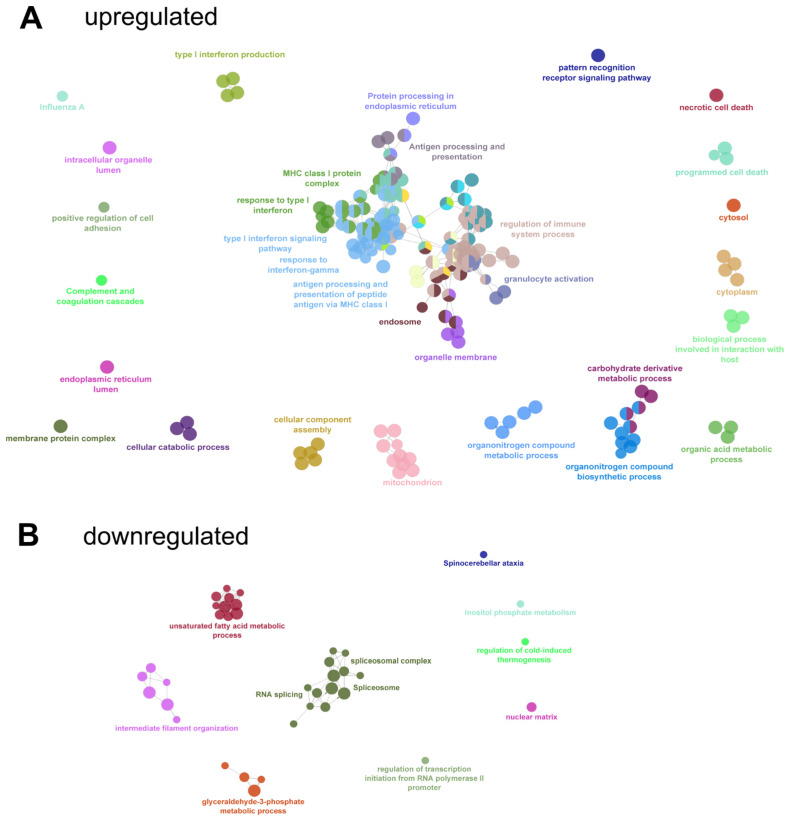
GO network analysis. Early (1 dpi, 2 dpi) and late (9 dpi, 12 dpi) stages were compared and upregulated (**A**) or downregulated (**B**) genes at late stages were subjected to GO-term enrichment analysis followed by network analysis with the ClueGO package of Cytoscape. Related GO-terms for clusters are labeled with one representative GO-term name from the cluster. Analysis parameters and detailed results can be found in Tables S2 and S3.

To get an overview of enriched GO and KEGG terms and their relation, enrichment analysis of DEG followed by network analysis of enriched terms was performed in Cytoscape (Figure 8, Tables S2 and S3). The analysis revealed the upregulation of multiple innate-immunity related pathways including type I and gamma IFN signaling and responses, antigen processing, MHC I protein complexes, and protein processing (Figure 8A), indicating a broad upregulation of anti-viral and immune system activating factors. In contrast, downregulated pathways included metabolic and mRNA splicing processes (Figure 8B).

This result was confirmed by comparative GO term enrichment analysis of differentially regulated genes with gProfiler software. Overexpressed genes in the late phase were annotated mainly to innate immune response processes (GO:0006955, GO:0002218, GO:0002252, GO:0002253). In contrast, genes downregulated in the late phase were mainly involved in RNA splicing and mRNA processing (KEGG:03040, GO:0006397, GO:0008380, GO:0000398) (Table S5).

Cellular components (annotated in the GO:CC branch of the Gene Ontology) with increased expression included the proteasome complex (GO:0000502), MHC I peptide loading complex (GO:0042824), ER (GO:0005783), and mitochondria (GO:0005739).

**Figure 9:**
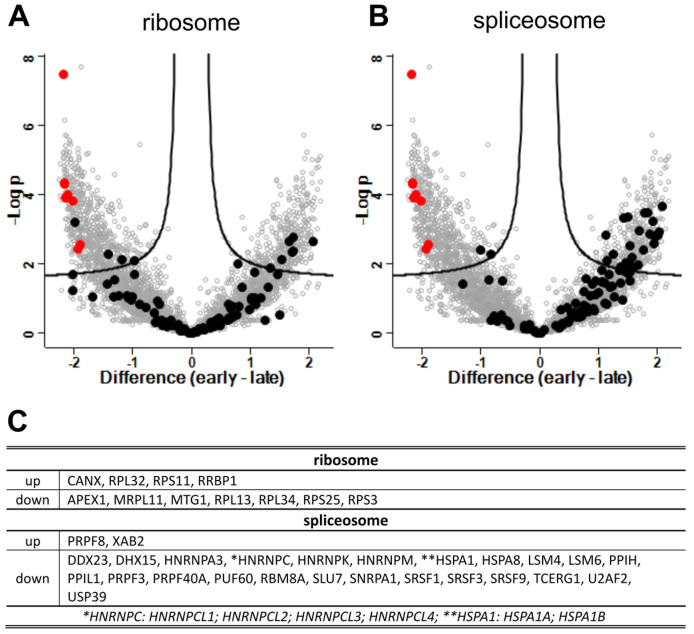
Differentially expressed genes (DEG) in late and early phases of infection: virus proteins, ribosome (KEGG:03010), and spliceosome (KEGG:03040). Volcano plots highlight genes annotated with GO terms (**A**) ribosome (KEGG:03010) and (**B**) spliceosome (KEGG:03040). NiV proteins are highlighted in red as reference for upregulated genes. Black dots represent cellular proteins of the respective pathways. (**C**) Regulated DEG corresponding to GO terms. *: No differentiation of the indicated HNRPNPCVs was possible. **: no differentiation of HSPA1A and HSPA1B was possible.

**Figure 10:**
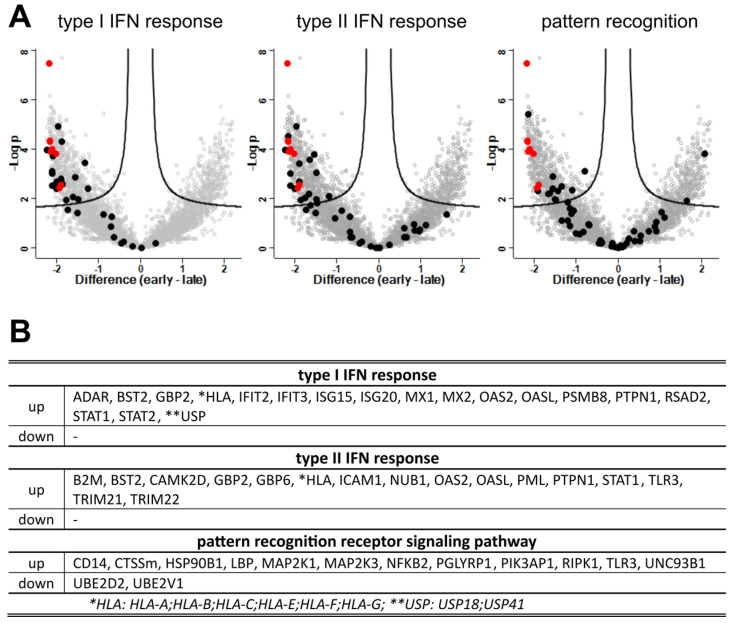
Differentially expressed genes (DEG) in late and early phases of infection: virus proteins, type I IFN response (GO: 0071357), and response to IFN gamma (GO:0034341). (**A**) Volcano plots highlight genes annotated within GO-terms response to Type I IFN (GO: 0071357), response to IFN gamma (GO:0034341), and the pattern recognition term GO:0002221. NiV proteins are highlighted in red as a reference for upregulated genes. Black dots represent cellular proteins of the respective pathways. (**B**) Regulated DEG corresponding to GO terms. *: No differentiation of the indicated HLAs was possible. **: no differentiation of USP18 and USP41 was possible.

**Figure 11:**
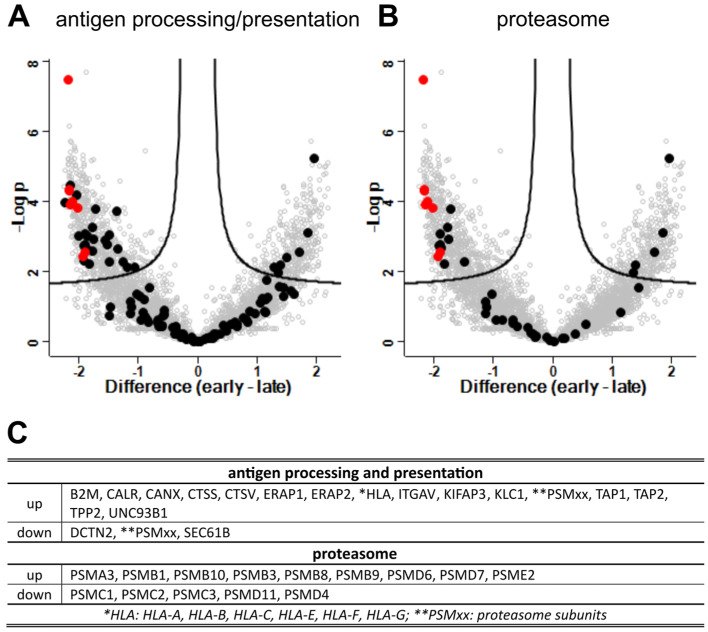
Differentially expressed genes (DEG) in late and early phases of infection: virus proteins, antigen processing and presentation (GO: 0048002), and proteasome (KEGG: 03050). (**A**) Volcano plots highlight genes in black related to the antigen processing and presentation GO term GO:0048002 and (**B**) the KEGG term Proteasome (KEGG:03050). NiV proteins are highlighted in red as a reference for upregulated genes. Black dots represent cellular proteins of the respective pathways. (**C**) Regulated DEG corresponding to GO terms. *: No differentiation of the indicated HLAs was possible. **: proteasomal subunits listed for proteasomes (KEGG: 03050).

Six GO/KEGG terms (Table 1) were selected for a more detailed analysis of the expression kinetics of the associated gene products. The relative expression levels of the proteins representing the respective GO terms were visualized in volcano plots in Figure 9, Figure 10 and Figure 11 together with ribosomal proteins as an unaffected control and NiV protein levels. For expression fold-change and *p*-values see Table S6.

While unaffected components such as the ribosomal proteins (Figure 9A) showed no significant changes in expression levels, a clear impact of the infection phase on the expression levels was observed for others. For instance, 23 proteins of the spliceosome complex were downregulated, while only two were upregulated (Figure 9B).

### 3.8. Interferons and Antigen Pattern Recognition

Upregulation of 24 proteins, including IFN-induced GTP-binding protein Mx proteins (MX1, MX2), 2′-5′-oligoadenylate synthases (OAS2, OASL), signal transducers and activators of transcription (STAT1, STAT2), and IFN-induced protein with tetratricopeptide repeats 2 (IFIT2) revealed strong induction of type I IFN signaling and response (Figure 10A). Elevated expression levels were detected for 16 proteins involved in type II IFN-gamma signaling, including beta-2-microglobulin (B2M), protein tyrosine phosphatase non-receptor type 1 (PTPN1), and E3 ubiquitin-protein ligases (TRIM22, TRIM22). These data indicated broad upregulation of type I and II IFN-related signaling throughout the NiV infection.

Accordingly, also 12 genes annotated within the pattern recognition signaling pathway (GO:0002221), including CD14, CTSS, MAP2K1, MAP2K3, NFKB2, RIPK1, and TLR3, were upregulated, whereas only the ubiquitin-conjugating factors UBE2D2 and UBE2V1 were decreased.

### 3.9. Antigen Processing and Presentation

Enrichment of proteins annotated with GO:0048002 (antigen processing and presentation of peptide antigen) and GO:0000502 (proteasome complex) also indicated potent upregulation of antigen processing and presentation upon virus infection (Figure 11). Of note, immunoproteasome subunit beta types PSMB8, PSMB9, and PSMB10, together with the PSME2 subunit of the immunoproteasome specific regulatory 11S cap structure, strongly indicated increased levels of immunoproteasomes with progressing virus infection. Concurrently decreased levels of PSMC1, PSMC3, PSMD4, and PSMD11, all part of the 19S regulatory subunit of constitutive proteasomes, further indicated conversion from conventional 26S proteasomes to immunoproteasomes.

Furthermore, increased levels of TAP-transporters (TAP1, TAP2), MHC I components B2M and HLAs, together with the ER chaperons calreticulin (CALR) and calnexin (CALN), and aminopeptidases ERAP1 and ERAP2 indicated upregulation of MHC I dependent antigen presentation with progressing infection. Of note, cathepsin S and V (CTSS, CTSV) levels also increased, indicating enhanced endosomal antigen processing and MHC I cross-presentation by the vacuolar pathway.

Overall, our data show that NiV infection in differentiated PBEC induces a robust innate immune response upon infection. We observed a strong IFN type I and II response together with upregulated antigen presentation.

## 4. Discussion

We analyzed the NiV infection and host reaction dynamics in differentiated primary PBEC-ALI cultures. By visualization of the infection course for 12 days after apical infection and correlation with quantitative high-resolution MS data, we provided insight into the dynamic and complex molecular virus-host interplay in an in vitro model for swine respiratory epithelia. In contrast to non-differentiated primary PBEC [13] and HBEC-ALI cultures [12], in the PBEC-ALI cultures, the NiV infection progressed slowly, was limited to laterally expanding foci (Figure 3), and induced multiple factors involved in type I and II IFN responses and other infection related pathways (Figure 10).

Previously described lack of efficient type I IFN response in NiV-infected HBEC-ALI cultures or in non-differentiated PBECs suggested a negligible role of type I IFN in NiV pathogenesis. Thus, low type I IFN levels were considered a prerequisite for efficient replication in respiratory epithelia and airborne virus spread [12,13]. However, our data show broad induction of anti-viral responses in fully differentiated PBEC-ALI with long term virus replication, virus spread, and strong CPE in the late phase of the infection. This aligns with IFN responses and subsequent expression of inflammation-related genes in henipavirus infected ferret lungs [15].

The low initial infection of PBEC-ALI cultures by NiV was not due to an immediate and broad type I IFN upregulation of host pathways identified in later phases, as IFN-β and IFN-λ mRNA levels increased later at 5 dpi (Figure 5). However, a rapid inhibitory response either directly by virus recognition or by ALI culture handling cannot be excluded, as mRNA levels of pro-inflammatory cytokines IL-6 and IL-8 were elevated directly after infection. Downregulation of both cytokines at 1 and 2 dpi and increased IFN- β, IFN-λ, IL-6, and IL-8 mRNA levels starting at 5 dpi correlated with the level of virus replication and protein expression (virus spread and RNA levels in Figure 2; NiV protein levels in Figure 6). Other reasons for low infection rates in the differentiated ALI-cultures could be a generally low susceptibility of the apical cell layers to NiV infection due to low receptor expression levels or a physical barrier generated by mucus secretion.

Although levels of viral proteins N, P, V, W, C, and M, involved in virus recognition and host response escape (reviewed in [14]), increased at 5 dpi, they were insufficient to suppress type I and II IFN and related host responses. Continuous spread in the PBEC-ALI cultures revealed the capability of the virus to replicate in the context of the observed host response, including ISGs inhibitory to multiple RNA viruses [33], such as BST2, ADAR, IFIT2/3, ISG15, ISG20, MX1, MX2, OAS2, and OASL (Figure 10).

Both, type I and II IFNs induce immunoproteasomes [34,35]. Immunoproteasomes are rapidly induced by IFN-γ treatment in respiratory cells in vitro and murine gammaherpesvirus-68 (MHV-68) lung infection [36]. IFN-γ and NF-κB dependent immunoproteasome upregulation was also observed for an NS1-deficient influenza A virus variant [37]. Induction of immunoproteasomes in PBEC-ALI cultures by NiV infection, as indicated by the increased expression of the 20S/11S subunits PSMB8, PSMB 9, PSMB 10, PSME2 and downregulation of the 19S subunits PSMC1, PSMC2, PSMC3, PSMD4, PSMD11 (Figure 11) was most likely IFN-driven. Concerted upregulation of immunoproteasomes, TAP1/2, MHC I subunits, related chaperones, endopeptidases ERAP1 and ERAP2, and lysosomal/endosomal cathepsins S and V proteases (Figure 11) pointed towards improved antigen processing and presentation of endogenous proteins.

Cathepsin S is expressed in antigen-presenting cells (APCs) and critical in the presenting exogenous antigens by MHC II [38]. Since the presence of APCs is excluded due to selection conditions for epithelial cells during PBEC preparation and culture [28] and lack of MHC II-related factors in the MS analysis, the cathepsins indicated increased protein degradation in endosomal compartments of epithelial cells. Indeed, cathepsin S is expressed in airway epithelial cells in an IFN-γ dependent manner [39,40], and higher cathepsin S levels might be a direct response to NiV related IFN-γ induction in the PBECs. Cathepsin S plays a vital role in generating TAP-independent cross-presentation [41] of MHC I in the vacuolar recycling pathway [42]. We demonstrated MHC I upregulation and hypothesized that NiV infection of bronchial epithelia leads to a potent MHC I presentation of TAP-dependent intracellular antigens and also contributes to the MHC I cross-presentation of lysosomal/endosomal NiV antigens in the absence of APCs [43].

Upregulation of the proteasome and peptide loading complex (PLC) after NiV infection have been described previously. However, selective upregulation of TAP1 and PSMB9 without PSMB8 and PSMB10 in endothelial cells was considered imbalanced [44]. Whereas our findings on type I and II IFN responses (Figure 10) are in line with IFN induction in endothelial cells [45], differences in the level of immunoproteasome and MHC- I upregulation suggest different capacities of endothelial cells and PBEC-ALI cultures to upregulate MHC I antigen presentation after NiV infection.

Overall, the host response pattern in the PBEC-ALI cultures with type I and II IFN signaling and downstream immunoproteasome-meditated MHC I antigen presentation suggests that infection of porcine bronchial epithelia not only leads to efficient NiV replication but also plays a crucial role in MHC I priming of adaptive immune responses to the virus. In the NiV infected pig, MHC I presentation may lead to both efficient CD8+ cytotoxic T cell (CTL) mediated elimination of infected cells and CTL-mediated immunopathogenesis in the lung. Notably, after aerosol infection of the African green monkey model, the severe respiratory disease went along with changes in cytokine response and activated CD8+ T cell numbers, but not with apparent neutralizing antibody titers over an 8–10 day course of disease [46].

Downregulation of RNA processing and metabolic pathways (Table S5) could be due to a general increase in CPE (Figure 3) or virus direct inhibition of cellular gene expression. Host manipulation by HeV and NiV is often discussed in the context of nuclear accumulation of the matrix protein M [47,48,49,50,51]. For example, binding of M to the treacle protein results in the silencing of rRNA biogenesis [52]. We did not observe an impact on rRNA biosynthesis, and thus could not confirm NiV M induced silencing of rRNA biogenesis. Homeostasis of most ribosomal proteins suggested constant levels of cellular protein synthesis over the 12 day infection period. Whether the downregulation of spliceosome components (Figure 9) was related to host manipulatory M or other NiV protein functions, or whether it was an indirect outcome of antiviral responses and their effects on spliceosomal mRNA modification remains to be clarified.

Marginal release of infectious virus at the apical and basal sides was observed. However, both virus RNA and protein levels were elevated in the cell samples, strongly indicating that the bulk of newly produced NiV remained cell-associated. Cell-associated measles virus (MeV) in human ALI cultures was recently shown to infect monocyte-derived macrophages as a first stage of infection in a new host. Moreover, dislodged infected epithelial patches and expulsion through coughing and sneezing were discussed to contribute to the high contagiosity of MeV by increasing the virus survival time and delivery of high infectious doses to the next host [53]. In contrast to MeV-infected human ALI cultures, where apical, ciliated cell patches were infected without signs of cell death, we observed strong cytopathic effects in the middle cell layers of the PBEC-ALI cultures. Together with the observed preservation of the apical cell layers as demonstrated by the integrity of cilia, tight-, and cell adherens junctions, this strongly suggests substantial differences in tropism and spread of MeV and NiV in differentiated ALI-cultures. However, we speculate that similar to the MeV model, airborne NiV spread is supported by the expulsion of cell debris and that inhalation of such material increases the local infectious dose after inhalation by the next host. Indeed, the bigger the exhaled particles are, the higher is the chance of containing an infectious virus [54].

**Figure 12:**
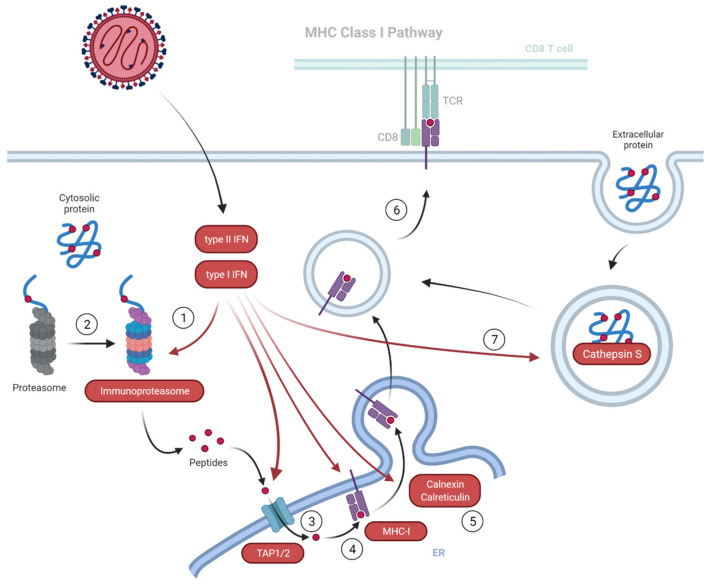
Model of type I/II IFN mediated upregulation of MHC I antigen presentation after NiV infection of PBEC-ALI cultures. Upon type I and type II interferon response (1), conventional proteasomes convert to immunoproteasomes (2). Simultaneously, upregulation of the TAP-heterodimer (3), MHC I subunits (4), and associated ER chaperons (5) indicate elevated peptide transport and MHC I antigen presentation (6). Elevated levels of endosomal protease cathepsin S (7) may lead to endosomal processing of extracellular antigens and cross-presentation through MHC I. Overall, in the bronchial epithelia, type I/II interferon responses may slow NiV replication and induce efficient processing of both intra- and extracellular antigens for priming of CD8+ T-cell mediated immune responses.

Based on the upregulation of the innate immune response and subsequent antigen proliferation and presentation pathways we propose a model in which NiV infection and spread in differentiated PBECs are slowed by potent innate immune responses to the virus infection (Figure 12). In contrast to previous reports in non-differentiated PBEC or endothelial cells, combining immunofluorescence, qRT-PCR and quantitative MS data, excludes limitations in IFN responses and incomplete immunoproteasome formation after NiV infection in fully differentiated PBEC. Altogether, our findings highlight the respiratory epithelium as a barrier to virus infections and indicate its role as a prime site of adaptive immune induction through NiV-induced antigen processing and MHC I presentation.

## Figures and Tables

**Table 1:** Overview of selected GO/KEGG terms for a more detailed analysis, based on GO-term enrichment analysis of differentially regulated genes with the gProfiler software (Table S5).

Regulation	Term Name	Go ID
up	antigen processing and presentation of peptide antigen	GO:0048002
up	response to type I IFN	KEGG: 03050
up	response to IFN gamma	GO:0071357
up	pattern recognition signaling pathway	GO:0034341
up	proteasome	GO:0002221
down	spliceosome	KEGG:03040

## Data Availability

The mass spectrometry proteomics data were deposited at the ProteomeXchange Consortium via the PRIDE partner repository with the dataset identifier PXD032673 and 10.6019/PXD032673.

## Supplementary Materials

The following supporting information can be downloaded at: https://www.mdpi.com/article/10.3390/v15040961/s1, Supplementary Material S1: methods [31,32,55,56,57,58,59,60,61,62,63,64,65,66,67,68,69,70]; Supplementary Material S2: primers and antibodies [13]; List S1: MQ parameters; Table S1: Gene list used for enrichment analysis; Table S2: CytoScape_ClueGO Parameters & Results downregulated; Table S3: CytoScape_ClueGO Parameters & Results upregulated; Table S4: Enrichment analysis_gProfiler_Neumann; Table S5: Enrichment analysis_gProfiler; Table S6: Summary_DEGs.
