# Supplementary material for: Analysis of Nipah Virus Replication and Host Proteome Response Patterns in Differentiated Porcine Airway Epithelial Cells Cultured at the Air–Liquid Interface
Source: Viruses. 2023 Apr 13;15(4):961. doi: 10.3390/v15040961 (PMC10143807; doi:10.3390/v15040961)
Supplement: Supplementary file 1 [file viruses-15-00961-s001.zip › List S1_ MQ parameters.pdf]

```
Parameter	Value
Version	2.0.2.0
User name	lc-user
Machine name	RIE-PC-N-20-054
Date of writing	08/31/2021 05:00:31
Include contaminants	True
PSM FDR	0.01
PSM FDR Crosslink	0.01
Protein FDR	0.01
Site FDR	0.01
Use Normalized Ratios For Occupancy	True
Min. peptide Length	7
Min. score for unmodified peptides	0
Min. score for modified peptides	40
Min. delta score for unmodified peptides	0
Min. delta score for modified peptides	6
Min. unique peptides	0
Min. razor peptides	1
Min. peptides	1
Use only unmodified peptides and	True
Modifications included in protein quantification	Oxidation (M);Acetyl (Protein N-term)
Peptides used for protein quantification	Razor
Discard unmodified counterpart peptides	True
Label min. ratio count	2
Use delta score	False
iBAQ	True
iBAQ log fit	True
Match between runs	False
Find dependent peptides	False
Fasta file	C:\pSEQ\EMBL_Sscr\NiV-Sscrofa11.1.2019-08-07-forpeaks.fasta
Decoy mode	revert
Include contaminants	True
Advanced ratios	True
Fixed andromeda index folder	
Combined folder location	F:\Data\A170.dda\MQ.test-2-samples
Second peptides	True
Stabilize large LFQ ratios	True
Separate LFQ in parameter groups	False
Require MS/MS for LFQ comparisons	True
Calculate peak properties	False
Main search max. combinations	200
Advanced site intensities	True
Write msScans table	False
Write msmsScans table	True
Write ms3Scans table	True
Write allPeptides table	True
Write mzRange table	True
Write DIA fragments table	True
Write DIA fragments quant table	True
Write pasefMsmsScans table	True
Write accumulatedMsmsScans table	True
Max. peptide mass [Da]	4600
Min. peptide length for unspecific search	8
Max. peptide length for unspecific search	25
Razor protein FDR	True
Disable MD5	False
Max mods in site table	3
Match unidentified features	False
Epsilon score for mutations	F:\Data\A170.dda\MQ.test-2-samples
```

Evaluate variant peptides separately True
Variation mode None
MS/MS tol. (FTMS) 20 ppm
Top MS/MS peaks per Da interval. (FTMS) 12
Da interval. (FTMS) 100
MS/MS deisotoping (FTMS) True
MS/MS deisotoping tolerance (FTMS) 7
MS/MS deisotoping tolerance unit (FTMS) ppm
MS/MS higher charges (FTMS) True
MS/MS water loss (FTMS) True
MS/MS ammonia loss (FTMS) True
MS/MS dependent losses (FTMS) True
MS/MS recalibration (FTMS) False
MS/MS tol. (ITMS) 0.5 Da
Top MS/MS peaks per Da interval. (ITMS) 8
Da interval. (ITMS) 100
MS/MS deisotoping (ITMS) False
MS/MS deisotoping tolerance (ITMS) 0.15
MS/MS deisotoping tolerance unit (ITMS) Da
MS/MS higher charges (ITMS) True
MS/MS water loss (ITMS) True
MS/MS ammonia loss (ITMS) True
MS/MS dependent losses (ITMS) True
MS/MS recalibration (ITMS) False
MS/MS tol. (TOF) 40 ppm
Top MS/MS peaks per Da interval. (TOF) 10
Da interval. (TOF) 100
MS/MS deisotoping (TOF) True
MS/MS deisotoping tolerance (TOF) 0.01
MS/MS deisotoping tolerance unit (TOF) Da
MS/MS higher charges (TOF) True
MS/MS water loss (TOF) True
MS/MS ammonia loss (TOF) True
MS/MS dependent losses (TOF) True
MS/MS recalibration (TOF) False
MS/MS tol. (Unknown) 20 ppm
Top MS/MS peaks per Da interval. (Unknown) 12
Da interval. (Unknown) 100
MS/MS deisotoping (Unknown) True
MS/MS deisotoping tolerance (Unknown) 7
MS/MS deisotoping tolerance unit (Unknown) ppm
MS/MS higher charges (Unknown) True
MS/MS water loss (Unknown) True
MS/MS ammonia loss (Unknown) True
MS/MS dependent losses (Unknown) True
MS/MS recalibration (Unknown) False
Site tables Oxidation (M)Sites.txt
