# Supplementary material for: Analysis of Nipah Virus Replication and Host Proteome Response Patterns in Differentiated Porcine Airway Epithelial Cells Cultured at the Air–Liquid Interface
Source: Viruses. 2023 Apr 13;15(4):961. doi: 10.3390/v15040961 (PMC10143807; doi:10.3390/v15040961)
Supplement: Supplementary file 1 [file viruses-15-00961-s001.zip › Supplementary Material S1_methods.pdf]

Preparation and cultivation primary porcine bronichal epithelial cells (PBEC). After revmoval of the lung, the bronchial tree was dissected, washed with phosphate-buffered saline (PBS) and cut into ~2 cm big pieces, followed by digestion in Dulbecco's Modified Eagle's Medium (DMEM, Gibco; Thermo Fisher, USA) supplemented with 1 mg/mL pronase (Roche, Switzerland), 10 µg/mL DNase I (AppliChem, Germany), 100 U/mL penicillin (Sigma-Aldrich, Germany), 100 µg/mL streptomycin (Sigma-Aldrich, Germany), 50 µg/mL gentamicin (Gibco; Thermo Fisher, USA), 1.25 µg/mL amphotericin B (Sigma-Aldrich, Germany) and 2 µg/mL fluconazole (Claris Lifesciences, UK) for 48 h at 4 °C with gentle shaking. Cells were harvested by scratching the inner site of the sections with a scalpel and expanded in cell culture flasks coated with collagen type I (PureCol; Advanced BioMatrix, USA) with PneumaCult-Ex medium (STEMCELL Technologies, Canada) at 37 °C and 5 % CO_2 until they reached 70-90 % confluency. The cells were then transferred to liquid nitrogen for storage. Cells were thawed at 37 °C and expanded in collagen type I coated flasks. 5 x 10^4 cells were transferred to collagen IV (Sigma-Aldrich) coated cell culture inserts (0.33 cm^2, polyethylenterephtalate [PET] membrane, 0.4 µm pore size; Corning, USA) and incubated at 37 °C and 5 % CO_2 with PneumaCult-Ex medium in both the basal and apical compartments insert until they reached confluency. The apical medium was then removed and the basal medium exchanged to PneumaCult-ALI medium (STEMCELL Technologies). To achieve complete mucociliary differentiation, the cells were cultured under air-liquid interface conditions for at least four weeks at 37 °C and 5 % CO_2. ALI medium in the basal compartment was exchanged every 2-3 days and the cell surface was washed once per week with Hanks′ Balanced Salt solution (HBSS).

Preparation and measurement of MS samples. Protein extracts from the TriFast peqGOLD workflow (extraction according to the manufacturer's protocol) were resuspended in 200 µl 20 mM Tris-HCl, pH 7.4, 50 mM DTT, 1 % SDS and incubated at 100°C for 10 min. After centrifugation for 10 min at 10.000 g and 4°C, the supernatants were collected, protein concentrations were determined densitometrically with Coomassie-stained SDS-polyacrylamide gels [1]. Aliquots containing 150 µg were FASP (filter aided sample preparation) digested with trypsin (Promega #V5111, Madison, USA) as described [2] with an enzyme to substrate ratio of 1:50 (w/w) and 30 kDa cut-off ultrafilters (Vivacon 500, Satorius #VN01H22, Goettingen, Germany). The resulting peptides were desalted using Pierce C18 tips (Thermo Fisher, USA), vacuum dried, and resuspended in 0.1 % formic acid (FA).

Per sample, 1 µg peptides were separated by nano reversed phase liquid chromatography using a nanoElute chromatography station together with an IonOpticks Aurora column (25 cm x 75

µm inside diameter, 1.6 µm particle size, C18 stationary phase) at a temperature of 40 °C with a flow rate of 0.4 µl/min. Solvent A was 0.1 % FA and solvent B 0.1 % FA in acetonitrile. Peptides were eluted with a 115 min gradient from 2 % to 16 % solvent B (0-60 min), 15-24 % solvent B (60-90 min), 24 %-34 % solvent B (90 min–105 min), 35-95 % solvent B (105-107 min) and 95 % solvent B (107-115 min). The TimsTOF Pro instrument (Bruker, Bremen, Germany) was equipped with a CaptiveSpray nano electrospray ion source and was operated in Parallel Accumulation and Serial Fragmentation (PASEF) mode using the standard method for proteome analysis (1.1sec cycle time) recommended by the manufacturer. The mass spectrometry proteomics data have been deposited to the ProteomeXchange Consortium (http://proteomecentral.proteomexchange.org) via the PRIDE partner repository [3] with the dataset identifier PXD032673 and DOI 10.6019/PXD032673.

Processing and analysis of MS data. Protein identification and label-free quantification (LFQ) were performed using MaxQuant (MQ) version 2.0.2.0 [4, 5] at standard conditions suggested for a timsTOF instrument (refer to List S1). The protein and peptide false discovery rates (FDR) were set to a maximum of 1 %. Oxidation of methionine and protein N-terminal acetylation were allowed as variable modifications, while carbamidomethylation of cysteine was set as fixed modification. Trypsin was chosen as protease, 2 missed cleavage sites were tolerated. A single sequence database was compiled from NiV (GenBank AF212302) and porcine (EMBL, S. scrofa version 11.1), protein sequences downloaded from Ensembl repository [6]. The resulting MQ table with identified and quantified protein groups were processed using in-house R scripts (version 4.0.3, [7]) together with R studio [8] before analysis in Perseus version 1.6.15.0 [9], with the workflow depicted in the figure 1 shown below. The R-packages gProfiler2 version 0.2.1 [10] and ggplot2 version 3.3.5 [11] were used to reference protein identifiers to genes and enrichment analysis or data visualization, respectively. The von Neumann test [12] was performed in R with package DescTools [13].

Tables for annotation of identified proteins and corresponding genes with experimental data (stages of infection, replicates), and data from public repositories KEGG (Kyoto Encyclopedia of Genes and Genomes) [14, 15] and GO (Gene Ontology) [16] were constructed in Perseus using in-house R scripts. KEGG and GO annotations of biological processes (GO:BP) and cellular components (GO:CC) were compiled from porcine annotations and annotations to the human orthologs of the identified porcine genes. For statistical analysis of Z-normalized gene expression levels in Perseus, the FDR was set to 5 % and S0 to 0 or 0.1 (default values) for t-tests and Volcano plots respectively.

Term enrichment analyses were performed as multi query based on up or down regulated differentially expressed genes (DEG) (see Table S1) using R together with the gProfiler2 package [10] or in Cytoscape version 3.9.1 [17] using the Plug-in ClueGO [18]. The detailed results can be found in table S2 and S3.

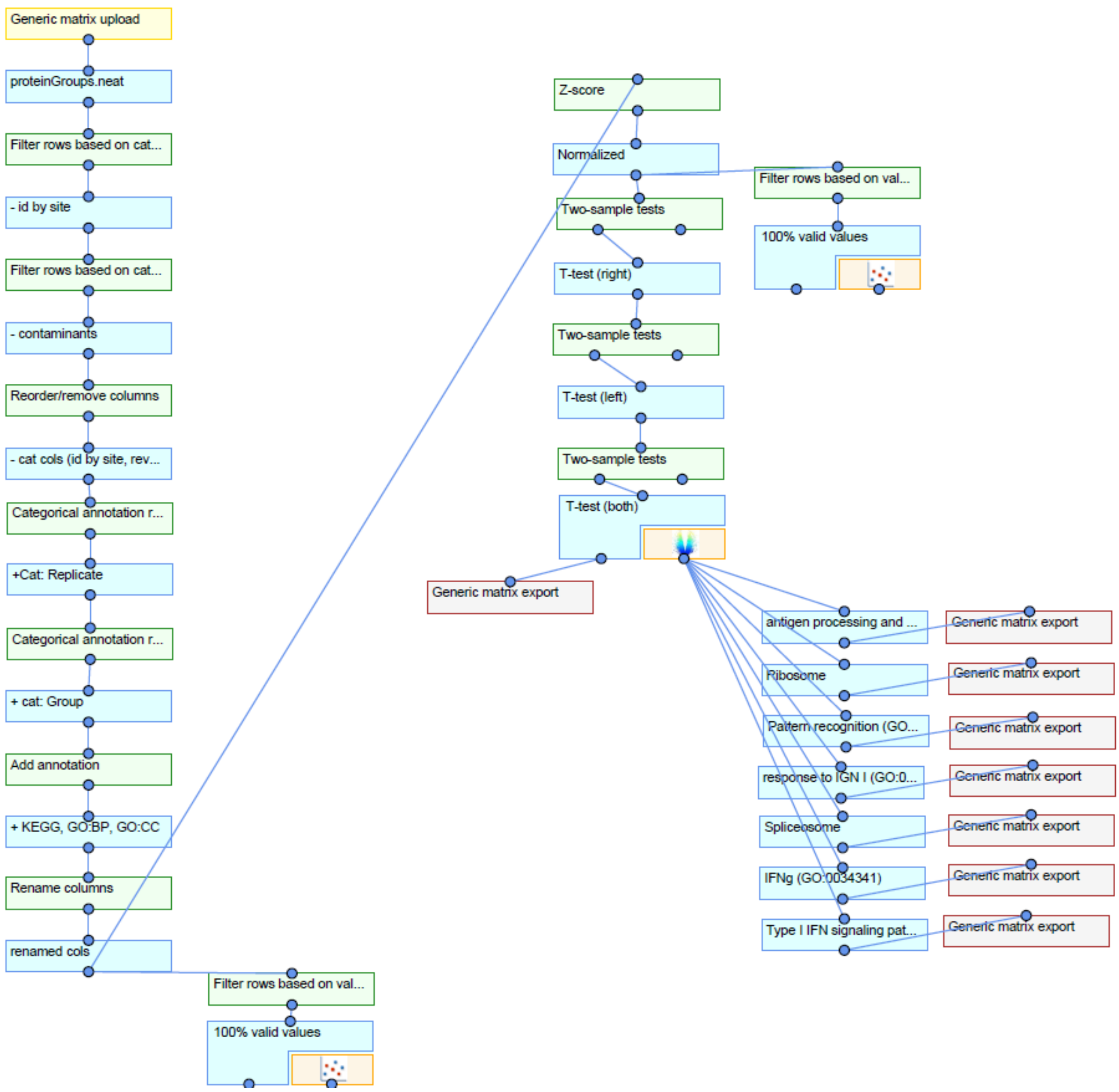

Figure S1. Workflow for the analysis in Perseus.

1. Neuhoff, V.; Arold, N.; Taube, D.; Ehrhardt, W., Improved staining of proteins in polyacrylamide gels including isoelectric focusing gels with clear background at nanogram sensitivity using Coomassie Brilliant Blue G-250 and R-250. *Electrophoresis* **1988,** 9, (6), 255-62.
2. Wisniewski, J. R.; Zougman, A.; Nagaraj, N.; Mann, M., Universal sample preparation method for proteome analysis. *Nat Methods* **2009,** 6, (5), 359-62.
3. Perez-Riverol, Y.; Csordas, A.; Bai, J.; Bernal-Llinares, M.; Hewapathirana, S.; Kundu, D. J.; Inuganti, A.; Griss, J.; Mayer, G.; Eisenacher, M.; Pérez, E.; Uszkoreit, J.; Pfeuffer, J.; Sachsenberg, T.; Yılmaz, Ş.; Tiwary, S.; Cox, J.; Audain, E.; Walzer, M.; Jarnuczak, A. F.; Ternent, T.; Brazma, A.; Vizcaíno, J. A., The PRIDE database and related tools and resources in 2019: improving support for quantification data. *Nucleic Acids Research* **2019,** 47, (D1), D442-D450.
4. Cox, J.; Mann, M., MaxQuant enables high peptide identification rates, individualized p.p.b.-range mass accuracies and proteome-wide protein quantification. *Nat Biotechnol* **2008,** 26, (12), 1367-72.
5. Prianichnikov, N.; Koch, H.; Koch, S.; Lubeck, M.; Heilig, R.; Brehmer, S.; Fischer, R.; Cox, J., MaxQuant Software for Ion Mobility Enhanced Shotgun Proteomics. *Mol Cell Proteomics* **2020,** 19, (6), 1058-1069.
6. Aken, B. L.; Achuthan, P.; Akanni, W.; Amode, M. R.; Bernsdorff, F.; Bhai, J.; Billis, K.; Carvalho-Silva, D.; Cummins, C.; Clapham, P.; Gil, L.; Giron, C. G.; Gordon, L.; Hourlier, T.; Hunt, S. E.; Janacek, S. H.; Juettemann, T.; Keenan, S.; Laird, M. R.; Lavidas, I.; Maurel, T.; McLaren, W.; Moore, B.; Murphy, D. N.; Nag, R.; Newman, V.; Nuhn, M.; Ong, C. K.; Parker, A.; Patricio, M.; Riat, H. S.; Sheppard, D.; Sparrow, H.; Taylor, K.; Thormann, A.; Vullo, A.;

Walts, B.; Wilder, S. P.; Zadissa, A.; Kostadima, M.; Martin, F. J.; Muffato, M.; Perry, E.; Ruffier, M.; Staines, D. M.; Trevanion, S. J.; Cunningham, F.; Yates, A.; Zerbino, D. R.; Flicek, P., Ensembl 2017. *Nucleic Acids Res* **2017,** 45, (D1), D635-D642.

7. R Core Team, *R: A language and environment for statistical computing. R Foundation for Statistical Computing*. Vienna, Austria, 2020.
8. RStudio Team, RStudio: Integrated Development for R. RStudio, PBC, Boston, Available at http://www.rstudio.com/. In 2020.
9. Tyanova, S.; Temu, T.; Sinitcyn, P.; Carlson, A.; Hein, M. Y.; Geiger, T.; Mann, M.; Cox, J., The Perseus computational platform for comprehensive analysis of (prote)omics data. *Nat Methods* **2016,** 13, (9), 731-40.
10. Kolberg, L., Raudvere, U., Package 'gprofiler2' Version 0.2.1. Available at https://cran.r-project.org/web/packages/gprofiler2/gprofiler2.pdf. In 2021.
11. Wickham, H. C., W.; Takahashi, K.; Wilke, C.; Woo, K.; Yutani, H.; Dunnington, D.; RStudio Package 'ggplot2' - Version 3.3.5. Available at https://cran.r-project.org/web/packages/ggplot2/ggplot2.pdf. In 2021.
12. von Neumann, J., Distribution of the Ratio of the Mean Square Successive Difference to the Variance. *The Annals of Mathematical Statistics* **1941,** 12, (4), 367-395, 29.
13. Andri et mult. al. S, DescTools: Tools for Descriptive Statistics. R package version 0.99.44, https://cran.r-project.org/package=DescTools. In 2021.
14. Kanehisa, M.; Goto, S., KEGG: kyoto encyclopedia of genes and genomes. *Nucleic Acids Res* **2000,** 28, (1), 27-30.
15. Kanehisa, M.; Furumichi, M.; Sato, Y.; Ishiguro-Watanabe, M.; Tanabe, M., KEGG: integrating viruses and cellular organisms. *Nucleic Acids Research* **2021,** 49, (D1), D545-D551.
16. Ashburner, M.; Ball, C. A.; Blake, J. A.; Botstein, D.; Butler, H.; Cherry, J. M.; Davis, A. P.; Dolinski, K.; Dwight, S. S.; Eppig, J. T.; Harris, M. A.; Hill, D. P.; Issel-Tarver, L.; Kasarskis, A.; Lewis, S.; Matese, J. C.; Richardson, J. E.; Ringwald, M.; Rubin, G. M.; Sherlock, G., Gene ontology: tool for the unification of biology. The Gene Ontology Consortium. *Nat Genet* **2000,** 25, (1), 25-9.
17. Shannon, P.; Markiel, A.; Ozier, O.; Baliga, N. S.; Wang, J. T.; Ramage, D.; Amin, N.; Schwikowski, B.; Ideker, T., Cytoscape: a software environment for integrated models of biomolecular interaction networks. *Genome research* **2003,** 13, (11), 2498-2504.
18. Bindea, G.; Mlecnik, B.; Hackl, H.; Charoentong, P.; Tosolini, M.; Kirilovsky, A.; Fridman, W.-H.; Pagès, F.; Trajanoski, Z.; Galon, J., ClueGO: a Cytoscape plug-in to decipher functionally grouped gene ontology and pathway annotation networks. *Bioinformatics* **2009,** 25, (8), 1091-1093.
