# Supplementary material for: Analysis of Nipah Virus Replication and Host Proteome Response Patterns in Differentiated Porcine Airway Epithelial Cells Cultured at the Air–Liquid Interface
Source: Viruses. 2023 Apr 13;15(4):961. doi: 10.3390/v15040961 (PMC10143807; doi:10.3390/v15040961)
Supplement: Supplementary file 1 [file viruses-15-00961-s001.zip › Supplementary Material S2_primers and antibodies.pdf]

Primer sequences for qPCR. Primer sequences are based on previous described work (Elvert et al., 2020).

Primer	Sequences
NiV-N for	5´-ATCAATCGTGGTTATCTTGA-3´
NiV-N rev	5´-CAGCCAGTTCTGCAACTTGATC-3´
porcine tubulin A1b for	5´-CTGAACCGCCTTATTAGCCAAA-3´
porcine tubulin A1b rev	5´-CGTTCAGGGCCCCATCA-3´
porcine IFN-β for	5´-GCTAACAAGTGCATCCTCCAAA-3´
porcine IFN-β rev	5´-AAGCACATCATAGCTCATGGAAAG-3´
porcine IFN-λ3 for	5´-AAGAGGGCCAAGGATGCCTTTGAA-3´
porcine IFN-λ3 rev	5´-AGGCGGAAGAGGTTGAACATGACA-3´
porcine OAS1 for	5´-GAGCTGCAGCGAGACTTCCT-3´
porcine OAS1 rev	5´-TGCTTGACAAGGCGGATGA-3´
porcine ISG56 for	5´-TCAGAGGTGAGAAGGCTGGT-3´
porcine ISG56 rev	5´-GCTTCCTGCAAGTGTCCTTC-3´
porcine IL-6 for	5´-TGTCGAGGCTGTGCAGATTAGT-3´
porcine IL-6 rev	5´-GGTGGCTTTGTCTGGATTCTTT-3´
porcine IL-8 for	5´-CCGTGTCAACATGACTTCCAA-3´
porcine IL-8 rev	5´-GCCTCACAGAGAGCTGCAGAA-3´

Elvert, M., L. Sauerhering, and A. Maisner. 2020. Cytokine Induction in Nipah Virus-Infected Primary Human and Porcine Bronchial Epithelial Cells. *J Infect Dis*. 221:S395-S400.

Primary antibodies

Target	Host species	Clonality	Source	RRID	Dilution
α-β-tubulin	Mouse	Monoclonal (TUB 2.1)	Sigma-Aldrich	AB_477577	1:100
α-Mucin-5AC	Mouse	Monoclonal (SPM297)	Origene	N/A	1:100
α-ZO-1	Rabbit	Polyclonal	Thermo Fisher	AB_2533938	1:200
α-β-catenin	Rabbit	Polyclonal	Thermo Fisher	AB_2533938	1:100
α-Cytokeratin 5	Rabbit	Polyclonal	Thermo Fisher	AB_869889	1:1000
α-HeV-N (detects NiV-N)	Rabbit	Polyclonal	FLI	N/A	1:1000
α-NiV-P	Rabbit	Polyclonal	FLI	N/A	1:1000

Fluorophore conjugated secondary antibodies

Target	Host species	Conjugate	Source	RRID	Dilution
α-rabbit IgG	Donkey	Alexa Fluor® 488	Invitrogen	AB_2535792	1:1000
α-mouse IgG	Donkey	Alexa Fluor® 488	Invitrogen	AB_141607	1:1000
α-rabbit IgG	Donkey	Alexa Fluor® 568	Invitrogen	AB_2534017	1:1000
α-mouse IgG	Donkey	Alexa Fluor® 568	Invitrogen	AB_2534013	1:1000
